# Evaluation of a modified venous excess ultrasound (VExUS) protocol for estimation of venous congestion: a cohort study

**DOI:** 10.1186/s13089-025-00411-x

**Published:** 2025-01-17

**Authors:** Katharine C. Martin, Edward A. Gill, Ivor J. Douglas, August A. Longino

**Affiliations:** 1 Department of Internal Medicine, University of Colorado Hospital, 12631 E 17thAvenue, Aurora, CO 80045 USA; 2 Department of Cardiology, University of Colorado Hospital, Aurora, CO USA; 3 Medicine, Pulmonary Sciences and Critical Care Medicine, University of Colorado Medical School and Denver Health Medical Center, Aurora, CO USA

## Abstract

**Background:**

Understanding venous congestion is critical to the management of many illnesses, but assessing volume status can be challenging. The current gold standard for volume status assessment of right heart catheterization (RHC) is invasive, costly, and often unavailable. Venous Excess Ultrasound Score (VExUS) is a novel ultrasound protocol for to assessment of venous congestion using the inferior vena cava, hepatic, portal and renal veins. Though there is a much interest in the technique, the renal component of the exam is challenging to acquire. For this reason we aimed to see if a modified VExUS (mVExUS) excluding the kidney component performs similarly to traditional VExUS (tVExUS) for detecting elevated right atrial pressure (RAP) as measured by RHC.

**Methods:**

A consecutive cohort of 95 patients undergoing RHC had VExUS exams before the procedure. Researchers compared the performance of tVExUS, mVExUS, and inferior vena cava (IVC) diameter in predicting RAP > 12 mmHg.

**Results:**

The area under the curve (AUC) for detecting elevated RAP was similar for tVExUS (0.87) and mVExUS (0.85). Both methods achieved high sensitivity and specificity. Agreement between tVExUS and mVExUS scores was near-perfect (Cohen's Kappa = 0.85).

**Conclusion:**

mVExUS may be as effective as tVExUS in identifying elevated RAP. This abbreviated version could improve efficiency and adoption of VExUS for assessing venous congestion. Further studies are needed in diverse patient populations.

**Supplementary Information:**

The online version contains supplementary material available at 10.1186/s13089-025-00411-x.

## Background

Venous congestion is increasingly recognized as a significant cause of morbidity and mortality in many highly morbid conditions [1–8], contributing to cardiorenal acute kidney injury (AKI), pulmonary edema, and organ hypoperfusion, among others [9]. For this reason, the ability of providers to rigorously assess venous congestion is critical to the daily management of a wide variety of patients. Unfortunately, evaluation of venous congestion is clinically challenging, and conventional exam techniques are often inadequate [10, 11]. For this reason, clinicians seeking definitive information on a patient’s degree of venous congestion often rely on right heart catheterization (RHC), the clinical gold standard for assessing venous hypertension [12]. However, RHC is an invasive and costly procedure that is not universally available, and is associated with a risk of patient complication as high as 1%, even in high-volume centers [13]. These limitations have led to an ongoing search for a non-invasive, economical, and reliable bedside procedure that can be used to assess a patient’s degree of venous congestion at the bedside [14].

To address this need, the Venous Excess Ultrasound Score (VExUS)—a novel ultrasonographic technique was designed to noninvasively assess venous congestion [15]. The VExUS technique leverages the fact that characteristic Doppler waveforms are associated with different degrees of venous congestion in the peripheral organs, and combines them into a unified assessment of venous circulation, including measurements of the inferior vena cava (IVC), hepatic, portal, and renal veins [15, 16]. The initial VExUS study reported an positive likelihood ratio of 6.37 for the development of cardiorenal acute injury (AKI) [15], and VExUS has been shown to have clinical utility in a variety of settings, including predicting resolution of cardiorenal AKI, and in evaluating volume status in the perioperative, intensive, care, and emergency settings [17, 18]. Importantly, a recent study demonstrated that VExUS grade is closely correlated with right atrial pressure, showing VExUS to have an AUC of 0.99 for the detection of a right atrial pressure (RAP) of > 12 mmHg. [19] The technique has generated considerable interest as a means to guide therapies, and is currently the subject of multi-center prospective trials (ClinicalTrials.gov Identifier: NCT06227702) [20]. One barrier to widespread adoption of VExUS is the difficulty of the renal component, prompting calls for validation of a modified protocol. For this reason, we compared a modified version of the VExUS score excluding renal imaging (mVExUS) to traditional VExUS (tVExUS) for detection of elevated RAP, as well as evaluating each VExUS component.

## Methods

A consecutive convenience cohort of patients undergoing ambulatory and inpatient RHC at a tertiary center in Denver, CO from 12/20/2022–3/1/2023 underwent VExUS examination immediately prior to RHC. Blinded VExUS examinations were conducted and tVExUS grade was determined as previously described [15, 19]. The mVExUS grade was determined by applying the same grading algorithm after removing renal images (appendix 2).

Ultrasonographers were internal and emergency medicine residents with institutional training in ultrasound, and were not part of the clinical team. All ultrasonographers completed a 4-h video series on VExUS developed by the Beaubien-Souligny group [21], before undergoing in-person training by an Emergency Medicine attending physician with a subspeciality training in ultrasonography familiar with the VExUS technique. Prior to analysis, one of the clinicians that developed the VExUS score reviewed a subset of scans by videoconference to assess image quality and confirm grading accuracy. VExUS results were graded and recorded before publication of RHC results.

### Statistical methods

Descriptive statistics for the cohort including demographics, past medical history, echocardiographic characteristics, and indication for RHC are displayed in Table 1. Continuous variables are described by median and interquartile range, and ordinal and categorical variables are described by number and percentage.

**Table 1:** Cohort Characteristics

	N = 95^1^
Age	62 (54, 70)
Sex	
Male	63 (66%)
Female	32 (34%)
Body Mass Index	28 (25, 35)
History of heart failure with reduced ejection fraction	47 (49%)
History of myocardial infarction	24 (26%)
History of COPD	29 (31%)
ESRD on HD	4 (5.1%)
History of pulmonary hypertension	36 (38%)
Charlson Comorbidity Index	4.00 (3.00, 6.00)
Mitral regurgitation	40 (43%)
Mitral regurgitation severity	
Mild	24 (60%)
Moderate	11 (28%)
Severe	5 (12%)
Mitral stenosis	1 (1.1%)
Mitral stenosis severity	
Mild	1 (100%)
Moderate	0 (0%)
Severe	0 (0%)
Aortic regurgitation	17 (18%)
Aortic regurgitation severity	
Mild	13 (76%)
Moderate	3 (18%)
Severe	1 (5.9%)
Aortic stenosis	4 (4.3%)
Aortic stenosis severity	
Mild	1 (25%)
Moderate	2 (50%)
Severe	1 (25%)
Tricuspid regurgitation	36 (39%)
Tricuspid regurgitation severity	
Mild	17 (47%)
Moderate	15 (42%)
Severe	4 (11%)
Tricuspid stenosis	0 (0%)
Tricuspid stenosis severity	
Mild	0 (NA%)
Moderate	0 (NA%)
Severe	0 (NA%)
Nagueh L atrial pressure	17 (13, 24)
tVExUS	
0	37 (39%)
1	29 (31%)
2	16 (17%)
3	13 (14%)
mVExUS	
0	38 (40%)
1	30 (32%)
2	18 (19%)
3	9 (9.5%)
Most recent ejection fraction	30 (20, 39)
Right heart catheterization indication	
Abnormal stress test	1 (1.1%)
Angina	13 (14%)
Cardiogenic shock	1 (1.1%)
Cardiomyopathy	3 (3.2%)
Chronic respiratory failure	1 (1.1%)
Combined heart failure	5 (5.3%)
Coronary artery disease	3 (3.2%)
Diastolic heart failure	3 (3.2%)
Dyspnea	5 (5.3%)
Hypoxemic respiratory failure	1 (1.1%)
NSTEMI	5 (5.3%)
Pericardial effusion	2 (2.1%)
Pericarditis	1 (1.1%)
Pulmonary hypertension	11 (12%)
Syncope	1 (1.1%)
Systolic heart failure	27 (28%)
Unspecified heart failure	4 (4.2%)
Valvular disease	6 (6.3%)
Volume overload	2 (2.1%)

*COPD* Chronic Obstructive Pulmonary Disease, *ESRD* End Stage Renal Disease, *HD* Hemodialysis, *mVExUS* Modified VExUS, *tVExUS* Traditional VExUS

^1^Median (IQR); n (%)

We constructed Receiver Operatic Characteristic (ROC) curves for tVExUS and mVExUS for prediction of RAP > 12 mmHg, as well as a continuous measurement of IVC diameter. We also evaluated each component of the VExUS exam: hepatic, portal, and renal Doppler, and a binary cutpoint of IVC diameter of 2 cm, We also calculated Cohen’s Kappa statistic for agreement between tVExUS and mVExUS for VExUS grade. We used Youden indexing to calculate cut-points for tVExUS and mVExUS to maximize sensitivity and specificity for a RAP > 12 mmHg. The threshold used for statistical significance was p < 0.05. Calculations were conducted using R version 4.2.1 (2022-06-23).

## Results

**Fig. 1:**
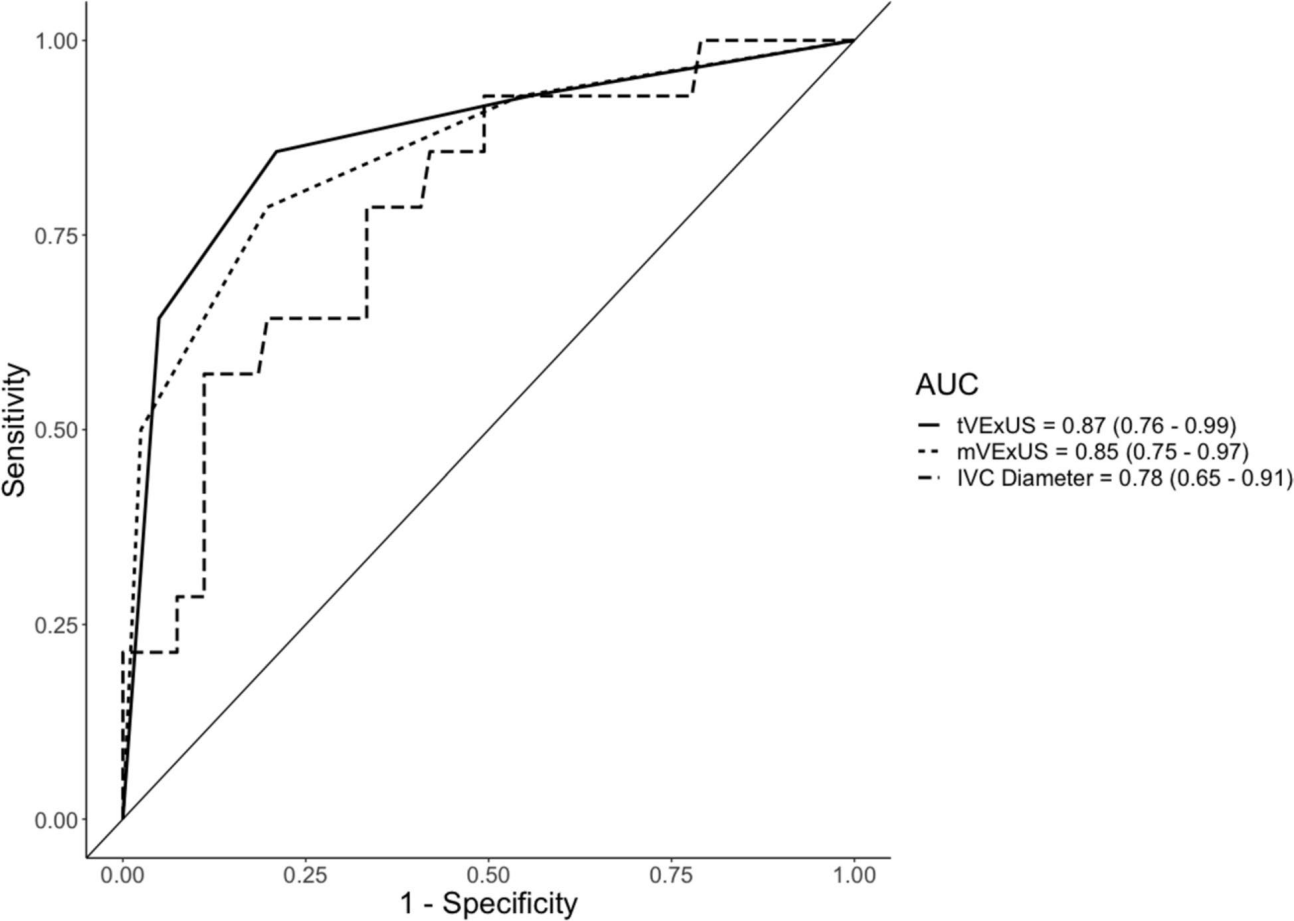
A comparison of receiver operating characteristic (ROC) curves for traditional and modified VExUS as well as IVC diameter. The area under the curve (AUC) for tVExUS, mVExUS, and IVC diameter were 0.87 95%CI (0.76–0.99), 0.85 95%CI (0.75–0.97) and 0.78 95%CI (0.65–0.91), respectively. A tVExUS grade of 3 had a sensitivity of 0.86 95%CI (0.62–1) and specificity of 0.79 95%CI (0.73–0.98), an mVExUS grade of 3 had a sensitivity of 0.79 95%CI (0.54–1) and specificity of 0.8 95%CI (0.71–0.99)

95 Patients were included in the study, 53 of which were inpatients. Descriptive characteristics for the cohort are displayed in Table 1. No patients required vasoactive medications or mechanical ventilation at the time of study procedures. After ROC analysis for detection of a RAP > 12 mmHg, the area under the curve (AUC) for tVExUS, mVExUS, and IVC Diameter were 0.87 95%CI (0.76–0.99), 0.85 95%CI (0.75–0.97) and 0.78 95%CI (0.65–0.91), respectively (Fig. 1). A tVExUS grade of 3 had a sensitivity of 0.86 95%CI (0.62–1) and specificity of 0.79 95%CI (0.73–0.98), an mVExUS grade of 3 had a sensitivity of 0.79 95%CI (0.54–1) and specificity of 0.8 95%CI (0.71–0.99). The Cohen’s Kappa statistic for agreement between mVExUS and tVExUS was 0.85 95%CI (< 0.05). The AUC for the hepatic vein was 0.81 (0.72–0.92), portal vein 0.86 (0.76–0.96), renal vein 0.9 (0.83–0.98). All were comparable to the overall VExUS exam, and higher AUC of the 2-cm IVC cutoff (0.71 (0.62–0.79)) (Supplemental Fig. 1) (Fig. 1).

## Discussion

The results of the current study suggest that mVExUS retains a high sensitivity and specificity for elevated RAP when compared with tVExUS, as documented in a previous study [22]. The Cohen’s Kappa statistic of 0.85 indicates near-perfect agreement between the two scores, suggesting that they could be interchangeable in practice. When comparing mVExUS to prior techniques for assessing venous congestion, the mVExUS score performs better than physical examination of the internal jugular vein, and [11] interestingly, both mVExUS and tVExUS have a greater AUC for detection of elevated RAP than the IVC alone, as published in recent studies [23–25]. Interestingly, each Doppler component of VExUS had an AUC comparable to the overall score itself, suggesting that even further-truncated protocols may be feasible, and that IVC diameter may be a less-useful diagnostic tool than previously appreciated. The study has several key limitations, most importantly the small size and relative homogeneity of the cohort. No patients had shock, altered mental status, were undergoing positive pressure ventilation, or required vasoactive medications, limiting study generalizability. Strengths include a uniquely well-characterized cohort of patients including invasive hemodynamics, rigorously trained ultrasonographers, and a robust image evaluation process. There are many patient populations that require further study, including cardiogenic shock, renal disease, portal hypertension, and severe valvular disease, among others, and care should be taken when applying mVExUS in these populations. Nonetheless, these results suggest that abbreviated VExUS protocols may be used to gather accurate data about venous congestion, improving efficiency of clinical providers and allowing for increased uptake of this novel, broadly-applicable technique.

## Supplementary Information

Supplementary Material 1. Fig. S1. The individual Doppler components of the tVExUS protocol had similar values of AUC to the overall score, and significantly higher than the AUC of the 2-cm IVC cutoff.

## Data Availability

Data are available on request.
