# Supplementary figures and images for: Evaluation of a modified venous excess ultrasound (VExUS) protocol for estimation of venous congestion: a cohort study
Source: Ultrasound J. 2025 Jan 17;17:7. doi: 10.1186/s13089-025-00411-x (PMC11748731; doi:10.1186/s13089-025-00411-x)

Receiver Operating Characteristic Curves for Right Atrial Pressure > 12 mmHg

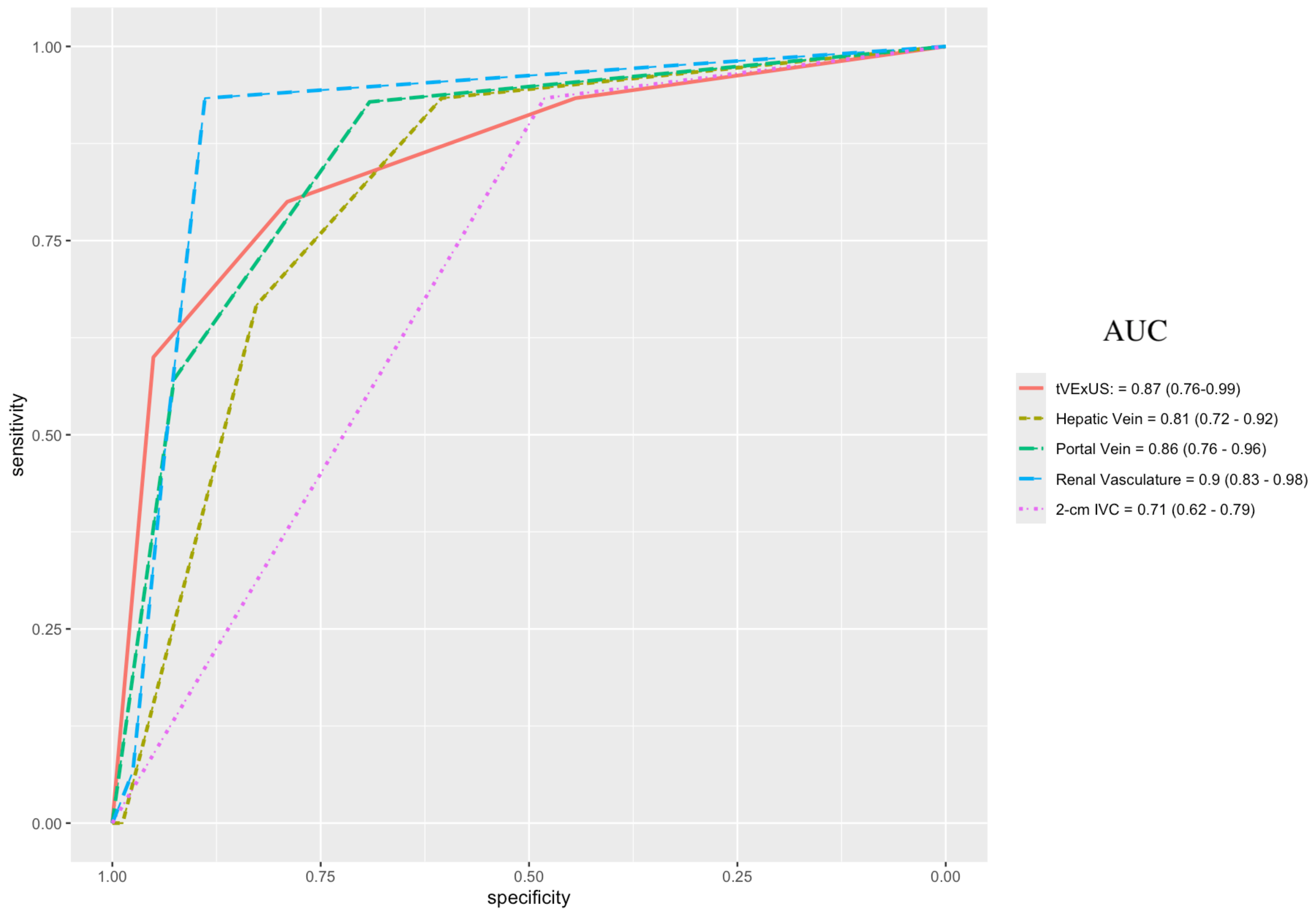

Supplement: Supplementary file 1 — Supplementary Material 1. Fig. S1. The individual Doppler components of the tVExUS protocol had similar values of AUC to the overall score, and significantly higher than the AUC of the 2-cm IVC cutoff. [file 13089_2025_411_MOESM1_ESM.pdf]
